# Supplementary material for: Multifunctional Properties of BMAP-18 and Its Aliphatic Analog against Drug-Resistant Bacteria
Source: Pharmaceuticals (Basel). 2023 Sep 25;16(10):1356. doi: 10.3390/ph16101356 (PMC10609797; doi:10.3390/ph16101356)
Supplement: Supplementary file 1 [file pharmaceuticals-16-01356-s001.zip › pharmaceuticals-2558194-supplementary.pdf]

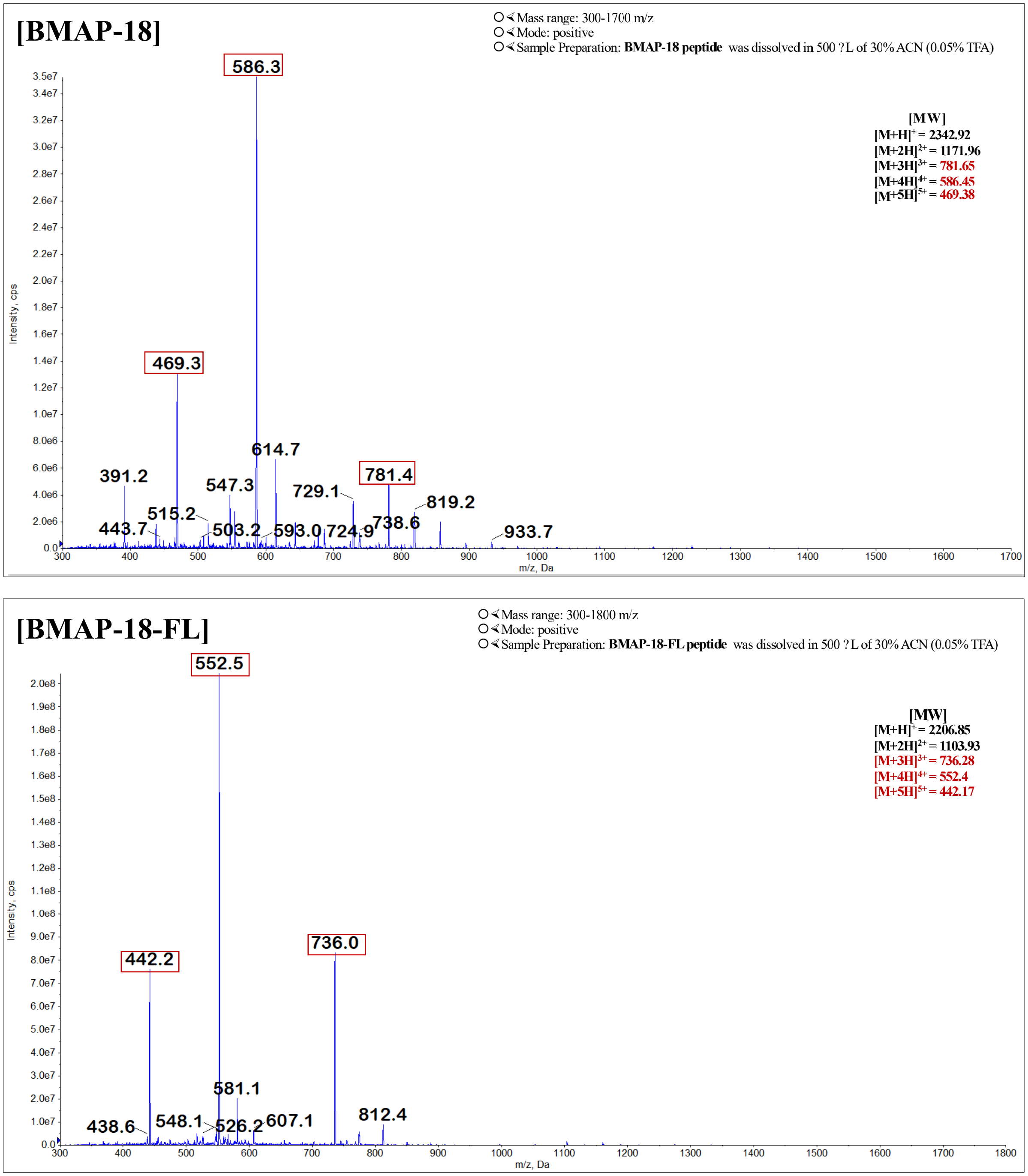


**Figure S1**. ESI-MS spectra of peptides. The molecular masses of BMAP-18 and BMAP-18-FL were analyzed using Electrospray Ionization-Mass Spectrometry (ESI-MS).

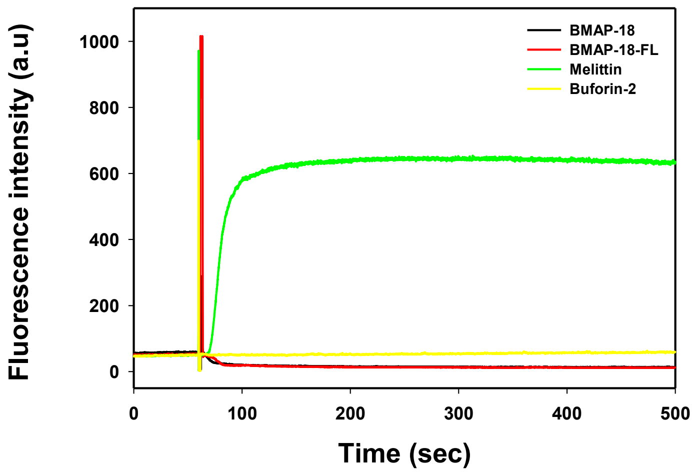


**Figure S2.** SYTOX green uptake caused by peptides. The SYTOX Green uptake assay was conducted on *S. aureus* with peptides administered at 1 MIC. SYTOX Green, a fluorescent nucleic acid stain, enters the bacterial cells through compromised membranes, leading to a detectable fluorescence signal. Melittin and buforin-2 were used as positive and negative controls, respectively. In comparison to melittin, both BMAP-18 and BMAP-18-FL showed no significant induction of SYTOX Green entry.

**SYTOX green uptake assay:** The effect of the peptides on bacterial membrane permeabilization was evaluated using the SYTOX green uptake assay of *S. aureus* (KCTC 1621) in mid-log phase. The culture was washed 3 times with a buffer (5 mM HEPES with 20 mM glucose). After washing, $10^6$ CFU/ml bacterial suspension was prepared and mixed with 0.5 μM SYTOX Green for 15 min in the dark. After the addition of peptides equal to 1 MIC concentration, the uptake of SYTOX Green was monitored using a Shimadzu RF-5300PC fluorescence spectrophotometer (Shimadzu Scientific Instruments, Japan), with wavelengths of 485 and 520 nm for excitation and emission, respectively. As control, one membrane disrupting peptide- melittin and one intracellular target peptide- buforin-2 were used for comparison.
